# The relationship between continuation of exercise habit for three years and endothelial function in patients with hypertension

**DOI:** 10.1038/s41440-024-02029-3

**Published:** 2024-12-05

**Authors:** Takayuki Yamaji, Farina Mohamad Yusoff, Shinji Kishimoto, Masato Kajikawa, Takahiro Harada, Aya Mizobuchi, Tatsuya Maruhashi, Ayumu Nakashima, Hirofumi Tomiyama, Yukihito Higashi

**Affiliations:** 1 Center for Radiation Disaster Medical Science, Research Institute for Radiation Biology and Medicine, Hiroshima University, Hiroshima, Japan; 2 Department of Regeneration and Medicine, Research Institute for Radiation Biology and Medicine, Hiroshima University, Hiroshima, Japan; 3 Division of Regeneration and Medicine, Medical Center for Translational and Clinical Research, Hiroshima University Hospital, Hiroshima, Japan; 4 Department of Nephrology, Graduate School of Medicine, University of Yamanashi, Yamanashi, Japan; 5 Department of Cardiology, Tokyo Medical University, Tokyo, Japan

**Keywords:** Exercise, Physical activity, Endothelial function, Flow-mediated vasodilation

## Abstract

The aim of this study was to evaluate the relationship between continuation of exercise habit for a long period and endothelial function assessed by flow-mediated vasodilation (FMD) in patients with hypertension. This study was a multicenter retrospective observational study. A total of 639 patients with hypertension were enrolled in this study. The subjects were divided into two groups based on information on exercise habit: a regular exercise group and a non-regular exercise group (control group). The regular exercise group was defined as patients who had an exercise habit during a 3-year follow-up period. There was no significant difference in FMD at baseline between the regular exercise group and control group. The change in FMD examined by the Wilcoxon signed rank test was significantly larger in the regular exercise group than in the control group (0.4 (−1.4, 2.0) % vs. −0.1 (−2.2, 1.4) %, *p* = 0.008). After adjustment for confounding factors for FMD, the odds ratio for increase in FMD was significantly larger in the regular exercise group than in the control groups. (OR: 1.59, 95% CI: 1.14–2.21, *p* = 0.006) A cubic spline curve revealed that even subjects with regular exercise who had a mean exercise intensity of less than 20 Mets・hour/week a had higher odds ratio for increase in endothelial function compared to the control group. These findings suggest that patients with hypertension who engage in regular exercise exhibited better endothelial function compared to those who do not exercise. Clinical Trial Registry Information: http://www.umin.ac.jp (UMIN000012951).

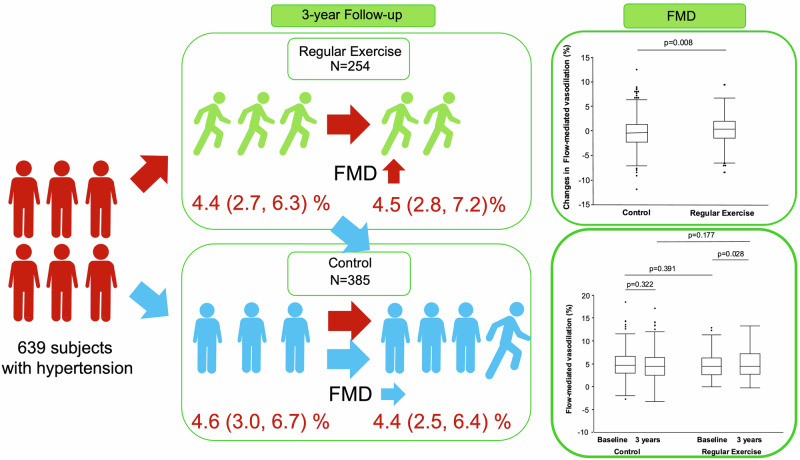

## Introduction

Hypertension is one of the most common diseases and one of the strongest risk factors for cardiovascular events and all-cause mortality [1]. More than 116 million people have hypertension in America and about 43 million people have hypertension in Japan. The number of patients with hypertension is continuing to incrase [2] and the cost of hypertension treatment is high. In America, the total cost for the treatment of hypertension was $59.9 billion per year [3].

Lifestyle modifications such as salt intake restriction, appropriate body weight control, alcohol intake restriction and exercise are useful for patients with hypertension to control blood pressure and to reduce the risk of cardiovascular events [4–7]. These lifestyle modifications can be started with no cost, as opposed to drug therapy. Several lines of evidence have shown that aerobic exercise training leads to a 5.4 mmHg reduction in systolic blood pressure [8], a 9% reduction in coronary heart disease and a 7% reduction in all-cause mortality [9]. Therefore, several guidelines including guidelines published by the World Health Organization, the American College of Cardiology, the American Heart Association, the European Society of Cardiology and the European Society of Hypertension recommend aerobic exercise, especially more than 75 minutes per week of moderate-intensity exercise [10–12].

Endothelial dysfunction is well known as the initial step for atherosclerosis [13]. Traditional cardiovascular risk factors cause endothelial dysfunction, finally leading to cardiovascular events [14]. Endothelial function is improved by pharmacological therapy [15], endovascular interventions [16], and lifestyle modifications [17]. Therefore, assessment of endothelial function is useful for estimating the risks for cardiovascular events. One of the most widely used tools for assessing endothelial function is measurement of flow-mediated vasodilation (FMD) in the brachial artery [18].

The benefit of aerobic exercise for decreasing cardiovascular risks including endothelial dysfunction is well established. A previous interventional study showed that moderate-intensity exercise for 12 weeks improves endothelial function [19], while the relationship between continuation of exercise habit for a long period and FMD are still unknown. Furthermore, it is well known that maintaining an exercise habit for a long period is more difficult than starting an exercise habit. Dishman et al. showed that about 50% of adults who began exercise stopped doing exercise within six month [20]. Sperandei et al. showed that 96% of new members of fitness centers stopped doing activities within 12 months [21].

Therefore, the aim of the present study was to evaluate the relationship between continuation of exercise habit for three years and FMD in patients with hypertension in a multicenter clinical trial.

Point of view

- Clinical relevance
  Patients with hypertension who engage in regular exercise exhibited better endothelial function compared to those who do not exercise. Establishment of a long-term exercise habit may contribute to increase in endothelial function.

- Future direction
  A long-term randomized interventional trial to examine the effect of exercise on endothelial function is warranted.
- Consideration for the Asian population
  The FMD-J study was conducted in Japan, located in East Asia. These results may contribute to promoting the establishment of long-term exercise habit as a treatment for hypertension in other Asian countries.

## Methods

### Study subjects

Between June 2010 and October 2015, a total of 966 subjects who had been under regular follow-up at any of the participating institutions for at least six months and with blood pressure of <150/95 mmHg were eligible for enrollment in Flow-mediated Dilation Japan (FMD-J) Study B. The exclusion criteria were as follows: subjects without information on exercise habit, subjects with severe chronic heart failure (New York Heart Association level of more than III), subjects with coronary heart disease, including a history of percutaneous coronary intervention or coronary bypass surgery, severe valvar heart disease, arrhythmia that requires treatment (i.e., atrial fibrillation, atrial flutter, permanent pacemaker implantation or frequent ventricular premature beats), malignancy, undergoing treatment with steroids, nonsteroidal anti-inflammatory drugs, or immunosuppressive drugs, history of stroke, aortic disease (except peripheral artery disease), or serious liver disease, and judgment of an attending physician that an individual is ineligible for inclusion in the study.

### Study design

This study was a retrospective multicenter observational cohort study that was conducted at 17 university hospitals in Japan to examine the usefulness of FMD assessment for management of patients with hypertension with a three-year follow-up period [22]. From a total of 966 subjects, 639 subjects were included after excluding subjects who were not followed up (*n* = 188), subjects with unclear images of the brachial artery for reliable FMD assessment (*n* = 104), subjects who did not have hypertension (*n* = 22), and subjects who were uncertain whether they would continue (*n* = 13) (Supplementary Fig. 1). This study was executed in accordance with the Good Practice Guidelines. All subjects gave written informed consent for participation in the study. The protocol was registered in the University Hospital Medical Information Network Clinical Trials Registry (UMIN000012951).

### Study procedures

Blood examinations and medical examinations were conducted at the start of the study and during a three-year follow-up period. The participants were managed by their attending physicians who were encouraged to treat cardiovascular risk factors including hypertension, dyslipidemia and diabetes mellitus.

### Measurements of blood samples and assessment of cardiovascular risk factors

The subjects fasted the previous night and abstained from drinking alcohol, smoking and consuming caffeine and vitamins for at least 12 hours prior to the study. Each subject was kept in the supine position in a quiet, dark, air-conditioned room (23 °C to 26 °C) throughout the study. A 23-gauge polyethylene catheter was inserted into the left antecubital vein. Hypertension was defined as current use of antihypertensive drugs or systolic blood pressure of more than 140 mm Hg or diastolic blood pressure of more than 90 mm Hg measured in a sitting position on at least 3 occasions [23]. Dyslipidemia was defined according to the third report of the National Cholesterol Education Program [24]. Diabetes mellitus was defined according to the American Diabetes Association recommendation [25]. The ethical committees of the participating institutions approved the study protocol. Written informed consent for participation in this study was obtained from all participants during the initial registration.

### Confirmation of exercise habit

Information on physical activity was obtained by using the self-reported modified Godin Leisure-Time Exercise questionnaire at baseline and 18 months and three years later [26]. This questionnaire consists of questions on exercise habit, frequency of exercise for more than 15 minutes each time (times/week), duration of exercise time (minutes), and types of exercise. The intensity of exercise was estimated on the basis of the type of exercise using the Compendium of Physical Activities [27]. We defined subjects with regular exercise as subjects who had 15 minutes or more of continuous exercise at least once a week and we defined the regular exercise group as having regular exercise during baseline and 3 years. We defined subjects with stop exercise as subjects who had an exercise habit at baseline but did not have an exercise habit either 18 months or 3 years later or both 18 months and 3 years later.

### Study protocol

This study was a retrospective observational study. Subjects were divided into two groups based on information on exercise habit: a regular exercise group and a non-regular exercise group (control group). The regular exercise group was defined as subjects with an exercise habit during a 3-year follow-up period. Blood sample, FMD, brachial ankle pulse wave velocity (baPWV) and information on exercise habits were measured at baseline and three years later. We assessed endothelial function by FMD and arterial stiffness by baPWV and examined changes in these parameters. After maintaining the supine position for 30 minutes, FMD and baPWV were measured. The observers were blind to the form of examination. Multivariate regression analysis was performed to identify independent variables associated with increase in FMD.

### Study 1. The relationship between continuation of regular exercise habit and FMD

In study 1, we assessed the relationship between continuation of an exercise habit for three years and FMD in 639 subjects with hypertension. We divided the subjects into two groups according to the continuation of exercise for three years.

### Study 2. The relationship between stopping exercise habit and FMD

In study 2, we assessed the relationship between termination of the exercise habit during the 3-year follow-up period and FMD in 356 subjects who had an exercise habit at baseline. We divided the subjects into two groups according to termination of the exercise habit during the 3-year period.

### Confirmation of exercise habit

Information on physical activity was obtained by using the self-reported modified Godin Leisure-Time Exercise questionnaire at baseline and 18 months and three years later [26]. This questionnaire consists of questions on exercise habit, frequency of exercise for more than 15 minutes each time (times/week), duration of exercise time (minutes), and types of exercise. The intensity of exercise was estimated on the basis of the type of exercise using the Compendium of Physical Activities [27].

### Measurements of FMD

A high-resolution linear artery transducer was coupled to computer-assisted analysis software (UNEXEF18G, UNEX Co., Nagoya, Japan) that used an automated edge detection system for measurement of the brachial artery diameter [28]. A blood pressure cuff was placed around the forearm of each subject. The brachial artery was scanned longitudinally 5 to 10 cm above the elbow. When the clearest B-mode image of the anterior and posterior intimal interfaces between the lumen and vessel wall was obtained, the transducer was held at the same point throughout the scan by using a special probe holder (UNEX Co.) to ensure consistency of the imaging. Depth and gain setting were set to optimize the images of the arterial lumen wall interface. When the tracking gate was placed on the intima, the artery diameter was automatically tracked, and the waveform of diameter changes over the cardiac cycle was displayed in real time using the FMD mode of the tracking system. This allowed the ultrasound images to be optimized at the start of the scan and the transducer position to be adjusted immediately for optimal tracking performance throughout the scan. Pulsed Doppler flow was assessed at baseline and during peak hyperemic flow, which was confirmed to occur within 15 seconds after cuff deflation. Blood flow velocity was calculated from the color Doppler data and was displayed as a waveform in real time. Baseline longitudinal images of the artery were acquired for 30 seconds, and then the blood pressure cuff was inflated to 50 mm Hg above systolic pressure for 5 minutes. The longitudinal image of the artery was recorded continuously until 5 minutes after cuff deflation. Pulsed Doppler velocity signals were obtained for 20 seconds at baseline and for 10 seconds immediately after cuff deflation. Changes in brachial artery diameter were immediately expressed as percentage change relative to the vessel diameter before cuff inflation. FMD was automatically calculated as the percentage change in peak vessel diameter from the baseline value. Percentage of FMD [(peak diameter—baseline diameter)/baseline diameter] was used for analysis. Blood flow volume was calculated by multiplying the Doppler flow velocity (corrected for the angle) by heart rate and vessel cross-sectional area (−r^2^). Reactive hyperemia was calculated as the maximum percentage increase in flow after cuff deflation compared with baseline flow. The correlation coefficient between FMD analyzed at the core laboratory and participant institutions was 0.84 (*p* < 0.001).

### Measurements of baPWV

baPWV was measured using a volume-plethysmographic apparatus (Form PWV/ABI, Omron Health Care Co, Kyoto, Japan). Four oscillometric cuffs were wrapped around both upper arms and lower legs. The cuffs were connected to an oscillometric pressure sensor for measurement of blood pressure and to a plethysmographic sensor for recordings of volume pulse form. Ankle-brachial pressure index value was automatically calculated by dividing the ankle systolic blood pressure of right and left sides by the higher brachial systolic blood pressure of either arm, and the lower value of ankle-brachial pressure index was used for analysis. baPWV was calculated automatically according to the following formula: baPWV =(D1-D2)/T, where D1 is the distance between the suprasternal notch and the ankle obtained by using the equation D1 = 0.8129×height (in cm) + 12.328, D2 is the distance between the suprasternal notch and the brachium obtained by using the equation D2 = 0.2195×height-2.0734, and T is the time interval between the wave front of the brachial waveform and that of the ankle waveform. The distance between sampling points of baPWV was calculated automatically by inputting the value of individual height.

### Statistical analysis

Numerical variables are expressed as means ± SD for parameters with normal distributions and as median (interquartile range) for parameters with skewed distributions. Normal distributions were evaluated using the Shapiro-Wilk test. All reported probability values were 2-sided, and a probability value of <0.05 was considered statistically significant. Categorical values were compared by means of the chi-square test. Continuous variables were compared by using Student’s *t*-test or Wilcoxon rank sum test. Comparisons between two related groups were carried out by the paired *t*-test or Wilcoxon signed-rank test. Multivariate logistic regression analysis was performed to identify independent variables associated with increase in FMD. Age, gender, body mass index, current smoking, presence of dyslipidemia, presence of diabetes mellitus, and high-sensitivity C-reactive protein (hsCRP) were entered into the multivariate logistic regression analysis. The effect of the exercise intensity on endothelial function was assessed as a continuous variable by cubic spline curves with the control group as the reference. All data were processed using JMP Pro. Ver 16.0 software (SAS Institute, Cary, NC, USA) and R software version 3.5.1 (R Foundation for Statistical Computing, Vienna, Austria).

## Results

### Study 1

#### Baseline characteristics of the subjects

The baseline characteristics of the 639 subjects with hypertension are summarized in the Supplementary Table 1. The mean age of the subjects was 62 ± 9 years. The 639 subjects included 360 men (56.3%). Among the subjects, 423 (66.4%) had dyslipidemia, 122 (19.1%) had diabetes mellitus and 73 (11.6%) were current smokers. The median FMD value was 4.5 (2.8, 6.5) % and the median baPWV value was 1594 (1415, 1779) cm/seconds.

#### Changes in clinical parameters after a 3-year follow-up period

**Fig. 1:**
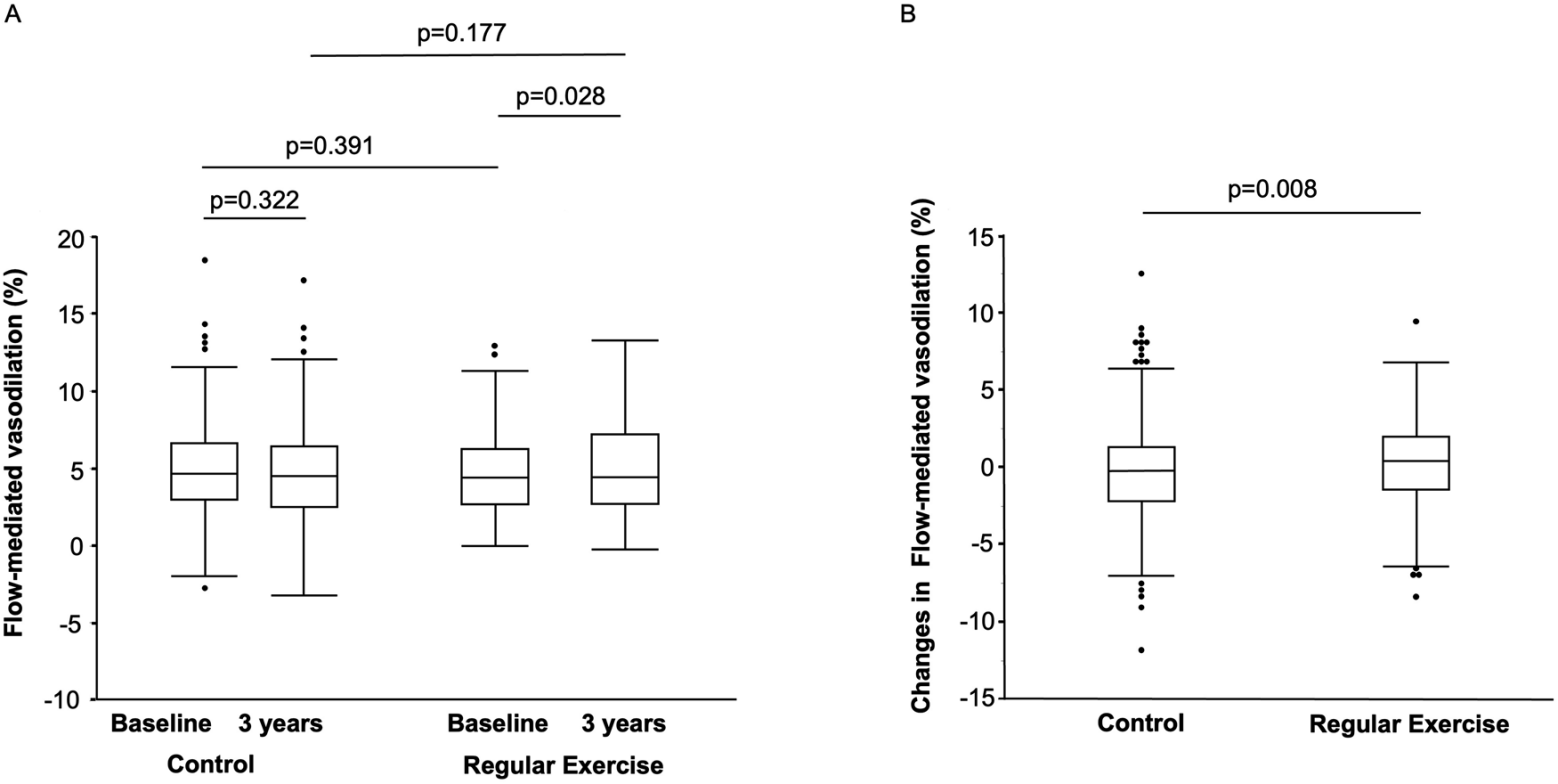
Bar graphs show flow-mediated vasodilation at baseline and after a 3-year follow-up period in patients with hypertension in the regular exercise group and the control group (**A**) and changes in flow-mediated vasodilation from baseline to the end of the 3-year follow-up period (**B**) examined by the Wilcoxon signed-rank sum test

Clinical parameters in the control group and in the regular exercise group are summarized in Table 1. At baseline, subjects in the regular exercise group were significantly older than those in the control group and BMI was significantly lower in the regular exercise group than in the control group at baseline. After the 3-year follow-up period, heart rate, diastolic blood pressure, total cholesterol, triglycerides and low-density lipoprotein cholesterol (LDL-C) were significantly decreased and creatinine was significantly increased in the regular exercise group compared to those in the control group. After the 3-year follow-up period, heart rate, systolic blood pressure, diastolic blood pressure, total cholesterol, triglycerides and LDL-C were significantly decreased and high-density lipoprotein cholesterol (HDL-C), creatinine and hemoglobin A1c were significantly increased in the control group. After the 3-year follow-up period, BMI was significantly lower in the regular exercise group than in the control group and systolic blood pressure was significantly higher in the regular exercise group than in the control group. FMD significantly increased from 4.4 (2.7, 6.3) % to 4.5 (2.8, 7.2) % in the regular exercise group (*p* = 0.028) but did not increase in the control group (4.6 (3.0, 6.7) % to 4.4 (2.5, 6.4) %, *p* = 0.322) (Fig. 1A). There was no change in baPWV after the 3-year follow-up in either group. The change in FMD was significantly larger in the regular exercise group than in the control group (0.4 (−1.4, 2.0) % vs. −0.1 (−2.2, 1.4) %, *p* = 0.008) (Fig. 1B), while changes in clinical parameters were similar in the two groups (Supplementary Table 2). Changes in baPWV (6.8 (−105, 127) vs. 29.5 (−128, 144) cm/seconds, *p* = 0.405) and changes in hsCRP level (16.5 (−199, 270) vs. 40.5 (−182, 362) mg/dL, *p* = 0.539) were similar in the two groups (Supplementary Table 2).

**Table 1:** Baseline clinical characteristics at baseline and after three years in patients with hypertension

	Control group		Regular exercise group	
Variables	Baseline (*n* = 385)	3 years (*n* = 385)	Baseline (*n* = 254)	3 years (*n* = 254)
Age, yr	61 ± 9		64 ± 8 *	
Men, *n* (%)	218 (56.6)		142 (55.9)	
Body mass index, kg/m^2^	25.0 ± 3.8	25.0 ± 4.0	24.2 ± 3.1*	24.1 ± 3.3*
Heart rate, bpm	72 ± 13	70 ± 13†	71 ± 11	69 ± 13†
Systolic blood pressure, mmHg	135 ± 15	133 ± 15 †	134 ± 15	135 ± 15*
Diastolic blood pressure, mmHg	80 ± 10	78 ± 11 †	80 ± 11	78 ± 11†
Total cholesterol, mmol/L	5.17 ± 0.93	5.04 ± 0.80 †	5.17 ± 0.85	5.04 ± 0.88 †
Triglycerides, mmol/L	1.13 (0.89, 1.84)	1.22 (0.88, 1.65) †	1.23 (0.85, 1.73)	1.19 (0.82, 1.64) †
HDL-C, mmol/L	1.50 ± 0.39	1.53 ± 0.39 †	1.53 ± 0.41	1.53 ± 0.41
LDL-C, mmol/L	3.00 ± 0.75	3.00 ± 0.72†	3.03 ± 0.78	3.00 ± 0.75†
Creatinine, µmol/L	68.07 ± 18.56	71.60 ± 29.17†	68.07 ± 17.68	70.72 ± 20.33†
Fasting blood glucose, mmol/L	5.83 ± 1.05	5.83 ± 1.28	5.77 ± 1.05	5.72 ± 0.94
Hemoglobin A1c, %	5.9 ± 0.6	6.0 ± 0.7†	5.9 ± 0.6	5.9 ± 0.6
hsCRP, ng/mL	480 (255, 1010)	506 (240, 1145)	489 (282, 908)	525 (284, 1040)
Medical history, *n* (%)				
Hypertension	385 (100)		254 (100)	
Dyslipidemia	254 (66.3)	257 (66.8)	169 (66.5)	180 (70.9)
Diabetes mellitus	77 (20.3)	82 (26.8)	45 (17.9)	48 (24.0)
Current Smoking, *n* (%)	50 (13.2)	44 (11.4)	23 (9.2)	21 (8.3)
Medication, *n* (%)				
Antihypertensive drugs	377 (97.9)	368 (95.6)	248 (97.6)	245 (96.5)
Calcium channel blockers	255 (66.2)	252 (65.5)	164 (64.6)	175 (68.9)
ARBs/ACEIs	255 (66.2)	265 (68.9)	176 (69.3)	160 (63.0)
Aldosterone antagonists	13 (3.4)	18 (4.7)	4 (1.6)	6 (2.4)
Lipid lowering drugs	140 (36.4)	157 (40.8)	84 (33.1)	101 (39.8)
Anti-diabetic drugs	47 (12.2)	55 (14.3)	20 (7.9)	24 (9.5)
baPWV, cm/seconds	1600 (1394, 2017)	1601 (1407, 1996)	1567 (1440, 1979)	1590 (1444, 2534)
FMD, %	4.6 (3.0, 6.7)	4.4 (2.5, 6.4)	4.4 (2.7, 6.3)	4.5 (2.8, 7.2)†

*HDL-C* high-density lipoprotein cholesterol, *LDL-C* low-density lipoprotein cholesterol, *hsCRP* high sensitivity C-reactive protein, *ARB* angiotensin receptor blocker, *ACE* angiotensin converting enzyme inhibitor, *baPWV* brachial ankle pulse wave velocity, *FMD* flow-mediated vasodilation

*P  < 0.05 vs Control group, †*P* <  0.05 vs Baseline

#### Multivariate analysis for the association between FMD and regular exercise

Multiple logistic analysis was carried out to determine whether the regular exercise group was associated with increase in FMD. The control group was used as a reference for deriving the increase in FMD in the regular exercise group. After adjustments for age, gender, BMI, presence of dyslipidemia, diabetes mellitus, current smoking, and hsCRP, the odds of having increase in FMD was significantly higher in the regular exercise group than in the control group (OR: 1.59, 95% CI: 1.14–2.21, *p* = 0.006) (Table 2).

**Table 2:** Multivariate analysis of relationships between increase in FMD and regular exercise

	Odds Ratio (95% Confidence Interval); *P* Value			
	Unadjusted	Model 1	Model 2	Model 3
Control	1 (reference)	1 (reference)	1 (reference)	1 (reference)
Regular exercise	1.56 (1.13–2.14); 0.007	1.61 (1.15–2.24); 0.005	1.60 (1.15–2.22); 0.005	1.59 (1.14–2.21); 0.006

Model 1; adjusted for age, gender, BMI, systolic blood pressure, triglyceride, LDL-C, fasting blood glucose, hsCRP, anti-hypertensive drug, anti-diabetic drug, lipid-lowering drug, and current smoking

Model 2; adjusted for age, gender, changes in BMI, changes in systolic blood pressure, changes in triglyceride, changes in LDL-C and changes in fasting blood glucose

Model 3; adjusted for age, gender, BMI, dyslipidemia, diabetes mellitus, current smoking, and hsCRP

*FMD* flow-mediated vasodilation, *BMI* body mass index, *LDL-C* low density lipoprotein cholesterol, *hsCRP* high sensitivity C-reactive protein

#### Exercise intensity and endothelial function

**Fig. 2:**
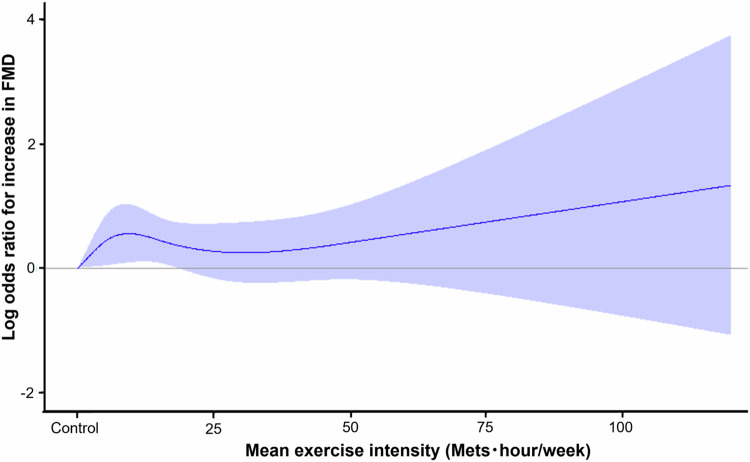
A cubic spline of exercise intensity with the log odds ratio for increase in flow-mediated vasodilation. Vertical lines show 95% confidence intervals

Next, we assessed the relationship between exercise intensity and FMD by cubic spline curves. The cubic spline curves revealed that even light-intensity exercise of less than 20 Mets・hour/week has a higher odds ratio for increase in FMD compared to the control group (Fig. 2).

### Study 2

#### Baseline characteristics of the subjects

At baseline, 356 subjects had an exercise habit. The baseline characteristics of the 356 subjects with hypertension and an exercise habit are summarized in Supplementary Table 3. The mean age of the subjects was 64 ± 8 years. The 356 subjects included 199 men (55.9%). Among the subjects, 239 (67.1%) had dyslipidemia, 68 (19.3%) had diabetes mellitus and 38 (10.8%) were current smokers. The median FMD value was 4.6 (2.9, 6.4) % and the mean baPWV value was 1607 (1441, 1791) cm/seconds.

#### Changes in clinical parameters after the 3-year follow-up period

**Fig. 3:**
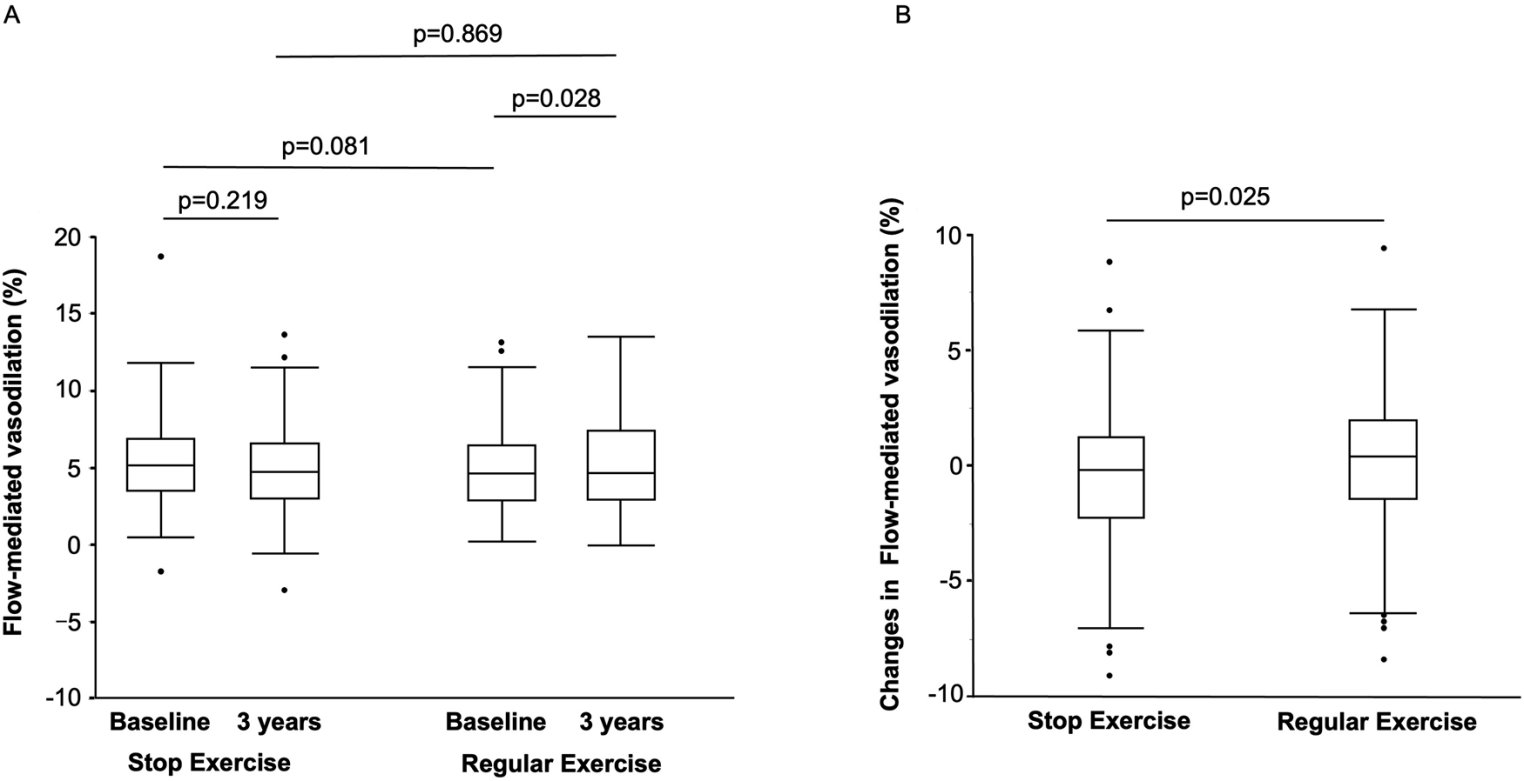
Bar graphs show flow-mediated vasodilation at baseline and after a 3-year follow-up period in patients with hypertension in the regular exercise group and the stop exercise group (**A**) and changes in flow-mediated vasodilation from baseline to the end of the 3-year follow-up period (**B**) examined by the Wilcoxon signed-rank sum test

We divided the subjects into two groups (stop exercise group and contentious regular exercise group). Clinical parameters in the stop exercise group and in the contentious regular exercise group are summarized in Supplementary Table 4. During the 3-year follow-up period, 102 subjects (28.7%) stopped doing exercise. At baseline, clinical parameters were similar in the stop exercise group and contentious regular exercise group. After the 3-year follow-up period, heart rate, diastolic blood pressure, total cholesterol, triglycerides and LDL-C and were significantly decreased and creatinine was significantly increased in the contentious regular exercise group. After the 3-year follow-up period, heart rate, systolic blood pressure, diastolic blood pressure, total cholesterol, triglycerides and LDL-C were significantly decreased and hemoglobin A1c was significantly increased in the stop exercise group. After the 3-year follow-up period, fasting blood glucose was significantly higher in the stop exercise group than in the contentious regular exercise group. FMD significantly increased from 4.4 (2.7, 6.3) % to 4.5 (2.8, 7.2), *p* = 0.028 in the contentious regular exercise group but did not increase in the stop exercise group (5.0 (3.3, 6.7) % to 4.5 (2.8, 6.4) %, *p* = 0.219) (Fig. 3A). baPWV was not altered after the 3-year follow-up period. The change in hemoglobin A1c was significantly larger in the stop exercise group than in the contentious regular exercise group (0.15 ± 0.60% vs. 0.01 ± 0.40%, *p* = 0.047) (Supplementary Table 5) and the change in FMD was significantly larger in the contentious regular exercise group than in the stop exercise group (0.4 (−1.4, 2.0) % vs. −0.2 (−2.2, 1.3) %, *p* = 0.025) (Fig. 3B). Changes in baPWV and changes in hsCRP level were similar in the two groups (Supplementary Table 5).

#### Multivariate analysis for the association between FMD and stopping exercise

The stop exercise group was used as a reference for deriving the increase in FMD in the contentious regular exercise group. After adjustments for age, gender, BMI, presence of dyslipidemia, diabetes mellitus, current smoking, and hsCRP, the odds of having increase in FMD was significantly higher in the contentious regular exercise group than in the stop exercise group (OR: 1.61, 95% CI: 1.01–2.59, *p* = 0.049) (Table 3).

**Table 3:** Multivariate analysis of relationships between increase in FMD and stopping exercise

	Odds Ratio (95% Confidence Interval); *P* Value			
	Unadjusted	Model 1	Model 2	Model 3
Stop exercise	1 (reference)	1 (reference)	1 (reference)	1 (reference)
Regular exercise	1.63 (1.03–2.59); 0.037	1.73 (1.06–2.80); 0.027	1.78 (1.11–2.86); 0.017	1.61 (1.01–2.59); 0.049

Model 1; adjusted for age, gender, BMI, systolic1blood pressure, triglyceride, LDL-C, fasting blood glucose, hsCRP, anti-diabetic drug, lipid-lowering drug, and current smoking

Model 2; adjusted for age, gender, changes in BMI, changes in systolic blood pressure, changes in triglyceride, changes in LDL-C and changes in fasting blood glucose

Model 3; adjusted for age, gender, BMI, dyslipidemia, diabetes mellitus, current smoking, and hsCRP.

*FMD* indicates flow-mediated vasodilation, *LDL-C* low density lipoprotein cholesterol, *BMI* body mass index, *hsCRP* high sensitivity C-reactive protein

## Discussion

In the present study, we assessed the changes in FMD in the regular exercise group and the control group in patients with hypertension. We demonstrated an association between regular exercise and better endothelial function than in those without exercise. Furthermore, 28.7% of the subjects who had an exercise habit stopped doing exercise during the 3-year follow-up period. The change in FMD after the 3-year follow-up period was significantly larger in the regular exercise group than in the stop exercise group. To our knowledge, the present study is the first study showing changes in FMD in the regular exercise group and the control group using real-world data on endothelial function assessed by FMD.

First, we assessed the changes in clinical parameters and FMD in the regular exercise group and the control group. Previous studies showed that exercise has beneficial effects on cardiovascular disease risk factors including body weight, blood pressure, triglycerides, LDL-C, and HDL-C [29–31]. In the present study, diastolic blood pressure, total cholesterol, triglycerides, and LDL-C were significantly decreased in the regular exercise group. However, changes in blood pressure, changes in triglycerides, changes in LDL-C and changes in glucose were similar in the control group and regular exercise group. There were no significant relationships between changes in FMD and changes in these parameters. It is unlikely that changes in these parameters contribute to the changes in FMD. In addition, after adjustment for confounding factors for endothelial function, the odds ratio for increase in FMD was significantly larger in the regular exercise group than in the control group. We also assessed the relationships between exercise intensity and FMD. A cubic spline curve revealed that subjects with exercise intensity of less than 20 Mets・hour/week increased FMD levels. Walking exercise corresponds to an exercise intensity of three Mets. Therefore, even subjects with walking for less than one hour per day for three years significantly increased FMD levels compared to subjects without exercise habit, suggesting the clinical significance of the establishment of an exercise habit.

Finally, we assessed the changes in clinical parameters and FMD in the regular exercise group and the stop exercise group. During the follow-up period, 28.7% of the subjects who had an exercise habit stopped doing exercise, and that percentage was lower than the percentages in previous studies [20, 21]. The change in FMD was significantly larger in the regular exercise group than in the stop exercise group. These findings suggest that discontinuation of exercise cancels the beneficial effects of exercise on endothelial function in patients with hypertension.

A possible mechanism by which exercise increase in FMD is an increase in nitric oxide (NO) bioavailability. Several lines of evidence have shown that exercise activity up-regulates NO bioavailability through an increase in NO production and reduction in the synthesis of asymmetric dimethylarginine, which is an inhibitor of endothelial NO synthase (eNOS) [32, 33]. It was previously shown that CRP per se downregulates eNOS activity in human aortic endothelial cells and in vivo in mice [34, 35]. However, in the present study, serum hsCRP did not change during the 3-year follow-up period in either the control group or the regular exercise group and serum hsCRP levels before and after the 3-year follow-up period were similar in the two groups. Our results suggest that regular exercise for, at least, three years has no anti-inflammatory effects in patients with hypertension.

There is still controversy about whether aerobic exercise improves arterial stiffness or not. Ashor et al. showed that aerobic exercise reduced PWV, while resistance exercise and combined exercise did not reduce PWV [36]. A meta-analysis revealed that aerobic exercise did not improve arterial stiffness assessed by PWV in patients with hypertension and subgroup analysis showed that exercise improved arterial stiffness when systolic blood pressure was reduced [37]. In the present study, arterial stiffness assessed by baPWV was not changed in the control group and regular exercise group after the 3-year follow-up period. We previously showed that 12-week regular exercise improved endothelial function assessed by vascular response to acetylcholine and an eNOS inhibitor but did not improve vascular smooth muscle function assessed by vascular response to sodium nitroprusside in patients with hypertension [19]. It is likely that aerobic exercise does not alter arterial stiffness and vascular smooth muscle function in patients with hypertension.

This study has several limitations. Although this study was conducted in multiple centers, the study was not a randomized trial. Because this was not a randomized interventional trial, this study cannot establish a cause-and-effect relationships between long-term exercise and changes in endothelial function. Second, this study was carried out in patients with hypertension. It is well known that patients with hypertension have an abnormal NO bioavailability [38, 39]. Assessment of the effects of regular exercise for healthy subjects on endothelial function would enable us to draw more specific conclusions concerning the role of exercise in endothelial function. Third, we did not have information on sitting time. Our previous study showed that sitting time on a non-working day was correlated with FMD and that FMD was smaller in subjects with sitting time on a non-working day of ≥six hours/day than in subjects with sitting time on a non-working day of <six hours/day [40]. We cannot deny the possibility that subjects without an exercise habit have longer sitting time and a longer sitting time, leading to endothelial dysfunction. Finally, unfortunately, in the present study, we did not have information on NO bioavailability and oxidative stress. Assessment of NO bioavailability and oxidative stress markers would enable more specific conclusions concerning the roles of long-term regular exercise in increase in endothelial function in patients with hypertension to be drawn.

### Perspective of Asia

Prevalence of hypertension is significantly higher in the Asian countries than the other countries. Furthermore, in Asia especially Japanese patients with hypertension, only about 30% achieve adequate blood pressure control, the rate was significantly lower in Western countries [41]. Several lines of evidence indicate that aerobic exercise training leads to blood pressure reduction [8, 11]. According to the World Health Organization (WHO), 31% of subjects worldwide are classified as physically inactive. However, the prevalence of physical inactivity in Asia is 48%, which exceeds that of other regions [42]. Not only blood pressure reduction, but exercise also has a reduction in coronary heart disease and reduction in all-cause mortality [9]. Therefore, increasing the prevalence of exercise habit in Asians is needed.

In conclusion, patients with hypertension who engage in regular exercise exhibited better endothelial function compared to those who do not exercise. Establishment of a long-term exercise habit may contribute to increase in endothelial function.

## Supplementary information

Supplementary Material

## References

[1] Kikuya M, Ohkubo T, Asayama K, Metoki H, Obara T, Saito S, et al. Ambulatory blood pressure and 10-year risk of cardiovascular and noncardiovascular mortality: the Ohasama study. Hypertension (Dallas, Tex : 1979). 2005;45:240–5. 15596571 10.1161/01.HYP.0000152079.04553.2c

[2] Umemura S, Arima H, Arima S, Asayama K, Dohi Y, Hirooka Y, et al. The Japanese Society of Hypertension Guidelines for the Management of Hypertension (JSH 2019). Hypertens Res. 2019;42:1235–481. 31375757 10.1038/s41440-019-0284-9

[3] Benjamin EJ, Muntner P, Alonso A, Bittencourt MS, Callaway CW, Carson AP, et al. Heart Disease and Stroke Statistics-2019 Update: A Report From the American Heart Association. Circulation. 2019;139:e56–e528. 30700139 10.1161/CIR.0000000000000659

[4] He FJ, Li J, Macgregor GA. Effect of longer term modest salt reduction on blood pressure: Cochrane systematic review and meta-analysis of randomised trials. Bmj. 2013;346:f1325. 23558162 10.1136/bmj.f1325

[5] Gay HC, Rao SG, Vaccarino V, Ali MK. Effects of Different Dietary Interventions on Blood Pressure: Systematic Review and Meta-Analysis of Randomized Controlled Trials. Hypertension (Dallas, Tex : 1979). 2016;67:733–9. 26902492 10.1161/HYPERTENSIONAHA.115.06853

[6] Roerecke M, Kaczorowski J, Tobe SW, Gmel G, Hasan OSM, Rehm J. The effect of a reduction in alcohol consumption on blood pressure: a systematic review and meta-analysis. Lancet Public Health. 2017;2:e108–e120. 29253389 10.1016/S2468-2667(17)30003-8 PMC6118407

[7] Casonatto J, Goessler KF, Cornelissen VA, Cardoso JR, Polito MD. The blood pressure-lowering effect of a single bout of resistance exercise: A systematic review and meta-analysis of randomised controlled trials. Eur J Prev Cardiol. 2016;23:1700–14. 27512052 10.1177/2047487316664147

[8] Saco-Ledo G, Valenzuela PL, Ruiz-Hurtado G, Ruilope LM, Lucia A. Exercise Reduces Ambulatory Blood Pressure in Patients With Hypertension: A Systematic Review and Meta-Analysis of Randomized Controlled Trials. J Am Heart Assoc. 2020;9:e018487. 33280503 10.1161/JAHA.120.018487 PMC7955398

[9] Whelton PK, He J, Appel LJ, Cutler JA, Havas S, Kotchen TA, et al. Primary prevention of hypertension: clinical and public health advisory from The National High Blood Pressure Education Program. Jama. 2002;288:1882–8. 12377087 10.1001/jama.288.15.1882

[10] Bull FC, Al-Ansari SS, Biddle S, Borodulin K, Buman MP, Cardon G, et al. World Health Organization 2020 guidelines on physical activity and sedentary behaviour. Br J Sports Med. 2020;54:1451–62. 33239350 10.1136/bjsports-2020-102955 PMC7719906

[11] Arnett DK, Blumenthal RS, Albert MA, Buroker AB, Goldberger ZD, Hahn EJ, et al. 2019 ACC/AHA Guideline on the Primary Prevention of Cardiovascular Disease: A Report of the American College of Cardiology/American Heart Association Task Force on Clinical Practice Guidelines. Circulation. 2019;140:e596–e646. 30879355 10.1161/CIR.0000000000000678 PMC7734661

[12] Bryan W, Giuseppe M, Wilko S, Enrico A R, Michel A, Michel Burnier et al. 2018 ESC/ESH Guidelines for the management of arterial hypertension. 2018;39:3021–104. 10.1093/eurheartj/ehy339 30165516

[13] Ross R. Atherosclerosis-an inflammatory disease. N. Engl J Med. 1999;340:115–26. 9887164 10.1056/NEJM199901143400207

[14] Maruhashi T, Soga J, Fujimura N, Idei N, Mikami S, Iwamoto Y, et al. Relationship between flow-mediated vasodilation and cardiovascular risk factors in a large community-based study. Heart. 2013;99:1837–42. 24153417 10.1136/heartjnl-2013-304739 PMC3841746

[15] Wolfrum S, Jensen KS, Liao JK. Endothelium-dependent effects of statins. Arterioscler Thromb Vasc Biol. 2003;23:729–36. 12615672 10.1161/01.ATV.0000063385.12476.A7

[16] Higashi Y, Sasaki S, Nakagawa K, Matsuura H, Oshima T, Chayama K. Endothelial function and oxidative stress in renovascular hypertension. N. Engl J Med. 2002;346:1954–62. 12075056 10.1056/NEJMoa013591

[17] Higashi Y, Sasaki S, Kurisu S, Yoshimizu A, Sasaki N, Matsuura H, et al. Regular aerobic exercise augments endothelium-dependent vascular relaxation in normotensive as well as hypertensive subjects: role of endothelium-derived nitric oxide. Circulation. 1999;100:1194–202. 10484540 10.1161/01.cir.100.11.1194

[18] Higashi Y. Assessment of endothelial function. History, methodological aspects, and clinical perspectives. Int Heart J. 2015;56:125–34. 25740586 10.1536/ihj.14-385

[19] Goto C, Higashi Y, Kimura M, Noma K, Hara K, Nakagawa K, et al. Effect of different intensities of exercise on endothelium-dependent vasodilation in humans: role of endothelium-dependent nitric oxide and oxidative stress. Circulation. 2003;108:530–5. 12874192 10.1161/01.CIR.0000080893.55729.28

[20] Dishman RK. Exercise Adherence–Its Impact on Public Health.' Champaign. IL: Human Kinetics Books; 1988).

[21] Sperandei S, Vieira MC, Reis AC. Adherence to physical activity in an unsupervised setting: Explanatory variables for high attrition rates among fitness center members. J Sci Med Sport. 2016;19:916–20. 26874647 10.1016/j.jsams.2015.12.522

[22] Tomiyama H, Kohro T, Higashi Y, Takase B, Suzuki T, Ishizu T, et al. A multicenter study design to assess the clinical usefulness of semi-automatic measurement of flow-mediated vasodilatation of the brachial artery. Int Heart J. 2012;53:170–5. 22790685 10.1536/ihj.53.170

[23] Shimamoto K, Ando K, Fujita T, Hasebe N, Higaki J, Horiuchi M, et al. The Japanese Society of Hypertension Guidelines for the Management of Hypertension (JSH 2014). Hypertens Res. 2014;37:253–390. 24705419 10.1038/hr.2014.20

[24] Expert Panel on Detection, Evaluation, and Treatment of High Blood Cholesterol in Adults. Executive Summary of The Third Report of The National Cholesterol Education Program (NCEP) Expert Panel on Detection, Evaluation, And Treatment of High Blood Cholesterol In Adults (Adult Treatment Panel III). Jama. 2001;285:2486–97. 10.1001/jama.285.19.2486 11368702

[25] American Diabetes Association. 2. Classification and Diagnosis of Diabetes: Standards of Medical Care in Diabetes-2019. Diabetes Care. 2019;42:S13–s28. 10.2337/dc19-S002 30559228

[26] Godin G, Shephard RJ. A simple method to assess exercise behavior in the community. Can J Appl Sport Sci. 1985;10:141–6. 4053261

[27] Ainsworth BE, Haskell WL, Herrmann SD, Meckes N, Bassett DR Jr, Tudor-Locke C, et al. 2011 Compendium of Physical Activities: a second update of codes and MET values. Med Sci Sports Exerc. 2011;43:1575–81. 21681120 10.1249/MSS.0b013e31821ece12

[28] Maruhashi T, Soga J, Fujimura N, Idei N, Mikami S, Iwamoto Y, et al. Nitroglycerine-induced vasodilation for assessment of vascular function: a comparison with flow-mediated vasodilation. Arterioscler Thromb Vasc Biol. 2013;33:1401–8. 23520168 10.1161/ATVBAHA.112.300934

[29] Lin X, Zhang X, Guo J, Roberts CK, McKenzie S, Wu WC, et al. Effects of Exercise Training on Cardiorespiratory Fitness and Biomarkers of Cardiometabolic Health: A Systematic Review and Meta-Analysis of Randomized Controlled Trials. J Am Heart Assoc. 2015;4:e002014. 10.1161/JAHA.115.002014 PMC4608087 26116691

[30] Snowling NJ, Hopkins WG. Effects of different modes of exercise training on glucose control and risk factors for complications in type 2 diabetic patients: a meta-analysis. Diabetes Care. 2006;29:2518–27. 17065697 10.2337/dc06-1317

[31] Kodama S, Tanaka S, Saito K, Shu M, Sone Y, Onitake F, et al. Effect of aerobic exercise training on serum levels of high-density lipoprotein cholesterol: a meta-analysis. Arch Intern Med. 2007;167:999–1008. 17533202 10.1001/archinte.167.10.999

[32] Vital TM, Stein AM, de Melo Coelho FG, Arantes FJ, Teodorov E, Santos-Galduróz RF. Physical exercise and vascular endothelial growth factor (VEGF) in elderly: A systematic review. Arch Gerontol Geriatr. 2014;59:234–9. 24856646 10.1016/j.archger.2014.04.011

[33] Riccioni G, Scotti L, Guagnano MT, Bosco G, Bucciarelli V, Di Ilio E, et al. Physical exercise reduces synthesis of ADMA, SDMA, and L-Arg. Front Biosci (Elite Ed). 2015;7:417–22. 25961421 10.2741/E739

[34] Venugopal SK, Devaraj S, Yuhanna I, Shaul P, Jialal I. Demonstration that C-reactive protein decreases eNOS expression and bioactivity in human aortic endothelial cells. Circulation. 2002;106:1439–41. 12234944 10.1161/01.cir.0000033116.22237.f9

[35] Schwartz R, Osborne-Lawrence S, Hahner L, Gibson LL, Gormley AK, Vongpatanasin W, et al. C-reactive protein downregulates endothelial NO synthase and attenuates reendothelialization in vivo in mice. Circ Res. 2007;100:1452–9. 17446434 10.1161/01.RES.0000267745.03488.47

[36] Ashor AW, Lara J, Siervo M, Celis-Morales C, Mathers JC. Effects of exercise modalities on arterial stiffness and wave reflection: a systematic review and meta-analysis of randomized controlled trials. PLoS One. 2014;9:e110034. 25333969 10.1371/journal.pone.0110034 PMC4198209

[37] Montero D, Roche E, Martinez-Rodriguez A. The impact of aerobic exercise training on arterial stiffness in pre- and hypertensive subjects: a systematic review and meta-analysis. Int J Cardiol. 2014;173:361–8. 24698257 10.1016/j.ijcard.2014.03.072

[38] Brandes RP. Vascular functions of NADPH oxidases. Hypertension (Dallas, Tex : 1979). 2010;56:17–21. 20479337 10.1161/HYPERTENSIONAHA.108.120295

[39] Gamil S, Erdmann J, Schwedhelm E, Bakheit KH, Abdalrahman IBB, Mohamed AO. Increased Serum Levels of Asymmetric Dimethylarginine and Symmetric Dimethylarginine and Decreased Levels of Arginine in Sudanese Patients with Essential Hypertension. Kidney Blood Press Res. 2020;45:727–36. 32814314 10.1159/000508695

[40] Yamaji T, Harada T, Hashimoto Y, Nakano Y, Kajikawa M, Yoshimura K, et al. Self-reported total sitting time on a non-working day is associated with blunted flow-mediated vasodilation and blunted nitroglycerine-induced vasodilation. Sci Rep. 2022;12:6366. 35430619 10.1038/s41598-022-10242-8 PMC9012897

[41] Kario K, Wang JG. Could 130/80 mm Hg Be Adopted as the Diagnostic Threshold and Management Goal of Hypertension in Consideration of the Characteristics of Asian Populations? Hypertension (Dallas, Tex : 1979). 2018;71:979–84. 29686008 10.1161/HYPERTENSIONAHA.118.11203

[42] Nearly 1.8 billion adults at risk of disease from not doing enough physical activity. 2024. https://www.who.int/news/item/26-06-2024-nearly-1.8-billion-adults-at-risk-of-disease-from-not-doing-enough-physical-activity). PMC11288492 39074895

