# Supplementary material for: The relationship between continuation of exercise habit for three years and endothelial function in patients with hypertension
Source: Hypertens Res. 2024 Dec 5;48(3):927–38. doi: 10.1038/s41440-024-02029-3 (PMC11879854; doi:10.1038/s41440-024-02029-3)
Supplement: Supplementary file 1 — Supplementary Material [file 41440_2024_2029_MOESM1_ESM.docx]

**Supplemental Material**

**The Relationship between Continuation of Exercise Habit for Three Years and Endothelial Function in Patients with Hypertension**

Short title: Regular exercise and endothelial function

Takayuki Yamaji, MD, PhD;^1,2^ Farina Mohamad Yusoff, MBBS, PhD, FSVM; ^2^ Shinji Kishimoto, MD, PhD;^2^ Masato Kajikawa, MD, PhD;^3^ Takahiro Harada, MD, PhD; ^2^ Aya Mizobuchi, MS;^2^ Tatsuya Maruhashi, MD, PhD; ^2^

Ayumu Nakashima, MD, PhD;^4^ Hirofumi Tomiyama, MD, PhD, FAHA;^5^

Yukihito Higashi, MD, PhD, FAHA^1,2,3^

^1^ Center for Radiation Disaster Medical Science, Research Institute for Radiation Biology and Medicine, Hiroshima University, Hiroshima, Japan

^2^ Department of Regeneration and Medicine, Research Institute for Radiation Biology and Medicine, Hiroshima University, Hiroshima, Japan

^3^ Division of Regeneration and Medicine, Medical Center for Translational and Clinical Research, Hiroshima University Hospital, Hiroshima, Japan

^4^ Department of Nephrology, Graduate School of Medicine, University of Yamanashi, Yamanashi, Japan

^5^ Department of Cardiology, Tokyo Medical University, Tokyo, Japan

Address for correspondence: Yukihito Higashi, MD, PhD, FAHA

Department of Cardiovascular Regeneration and Medicine,

Research Institute for Radiation Biology and Medicine, Hiroshima University

1-2-3 Kasumi, Minami-ku, Hiroshima 734-8551, Japan

Phone: +81-82-257-5831 Fax: +81-82-257-5831

E-mail: [yhigashi@hiroshima-u.ac.jp](mailto:yhigashi@hiroshima-u.ac.jp)

**Supplemental Tables**

**Supplementary Table 1.** Clinical Characteristics of Patients at Baseline and after Three Years

| Variables | Baseline  (n=639) | 3 years  (n=639) |
| --- | --- | --- |
| Age, yr | 62±9 |  |
| Men, n (%) | 360 (56.3) |  |
| Body mass index, kg/m^2^ | 24.7±3.5 | 24.6±3.8 |
| Heart rate, bpm | 72±12 | 70±13 † |
| Systolic blood pressure, mmHg | 134±15 | 134±15 |
| Diastolic blood pressure, mmHg | 80±10 | 78±11 † |
| Total cholesterol, mmol/L | 5.17±0.83 | 5.04±0.83 † |
| Triglycerides, mmol/L | 1.29 (0.82, 1.81) | 1.21 (0.87, 1.65) † |
| HDL-C, mmol/L | 1.50±0.41 | 1.53±0.39 † |
| LDL-C, mmol/L | 3.03±0.78 | 2.90±0.75 † |
| Creatinine, µmol/L | 68.07±17.68 | 70.72±26.52 † |
| Fasting blood glucose, mg/dL | 105±19 | 105±21 |
| Hemoglobin A1c, % | 5.9±0.6 | 5.9±0.6 |
| hsCRP, ng/mL | 487 (265, 962) | 514 (255, 1070) |
| Medical history, n (%) |  |  |
| Hypertension | 639 (100) |  |
| Dyslipidemia | 423 (66.4) | 437 (68.4) |
| Diabetes mellitus | 122 (19.1) | 130 (20.3) |
| Current Smoking, n (%) | 73 (11.6) | 65 (10.2) |
| Medication, n (%) |  |  |
| Antihypertensive drugs | 625 (97.8) | 613 (96.0) |
| Calcium channel blockers | 419 (65.6) | 427 (66.8) |
| ARBs/ACEIs | 431 (67.4) | 425 (66.5) |
| Aldosterone antagonists | 17 (2.7) | 24 (3.8) |
| Lipid lowering drugs | 224 (35.1) | 258 (40.3) |
| Anti-diabetic drugs | 67 (10.5) | 79 (12.4) |
| baPWV, cm/seconds | 1594 (1415, 1776) | 1595 (1427, 1802) |
| Flow-mediated vasodilation, % | 4.5 (2.8, 6.5) | 4.5 (2.6, 6.6) |

†P<0.05 vs Baseline

HDL-C indicates high-density lipoprotein cholesterol; LDL-C, low-density lipoprotein cholesterol; hsCRP, high sensitivity C-reactive protein; ARB, angiotensin receptor blocker; ACE, angiotensin converting enzyme inhibitor; baPWV, brachial ankle pulse wave velocity.

**Supplementary Table 2.** Changes in Clinical Characteristics of Patients in Two Groups of Exercise Habit

| Variables | Total | Control | Regular exercise | P value |
| --- | --- | --- | --- | --- |
| Changes in body mass index, kg/m^2^ | -0.07±1.6 | -0.02±1.8 | -0.1±1.4 | 0.346 |
| Changes in heart rate, bpm | -2.2±10.6 | -2.4±10.6 | -1.8±10.6 | 0.548 |
| Changes in systolic blood pressure, mmHg | -0.7±17.5 | -1.8±18.0 | 1.0±16.8 | 0.053 |
| Changes in diastolic blood pressure, mmHg | -1.1±9.9 | -1.1±9.6 | -1.1±10.4 | 0.913 |
| Changes in total cholesterol, mmol/L | -0.13±0.78 | -0.13±0.82 | -0.14±0.71 | 0.758 |
| Changes in triglycerides, mmol/L | -0.07 (-0.36, 0.19) | -0.09 (-0.40, 0.17) | -0.03 (-0.32, 0.24) | 0.146 |
| Changes in HDL-C, mmol/L | 0.02±0.21 | 0.03±0.22 | 0.01±0.20 | 0.136 |
| Changes in LDL-C, mmol/L | -0.11±0.70 | -0.12±0.74 | -0.11±0.65 | 0.922 |
| Changes in creatinine, µmol/L | 3.54±17.68 | 3.54±21.22 | 2.65±8.84 | 0.711 |
| Changes in fasting blood glucose, mmol/L | -0.02±1.01 | 0.01±1.08 | -0.06±0.91 | 0.473 |
| Changes in hemoglobin A1c, % | 0.03±0.4 | 0.04±0.4 | 0.01±0.4 | 0.424 |
| Changes in hsCRP, ng/mL | 23.5 (-188, 308) | 16.5 (-199, 270) | 40.5 (-182, 362) | 0.539 |
| Changes in baPWV, cm/seconds | 14 (-114, 132) | 6.8 (-105, 127) | 29.5 (-128, 144) | 0.405 |
| Changes in flow-mediated vasodilation, % | 0.1 (-1.9, 1.8) | -0.1 (-2.2, 1.4) | 0.4 (-1.4, 2.0) | 0.008 |

HDL-C indicates high-density lipoprotein cholesterol; LDL-C, low-density lipoprotein cholesterol; hsCRP, high sensitivity C-reactive protein; baPWV, brachial ankle pulse wave velocity.

**Supplementary Table 3.** Clinical Characteristics of Patients who had Exercise Habit at Baseline

| Variables | Baseline  (n=356) | 3 years  (n=356) |
| --- | --- | --- |
| Age, yr | 64±8 |  |
| Men, n (%) | 199 (55.9) |  |
| Body mass index, kg/m^2^ | 24.4±3.2 | 24.2±3.3 † |
| Heart rate, bpm | 71±12 | 69±13 † |
| Systolic blood pressure, mmHg | 135±15 | 135±15 |
| Diastolic blood pressure, mmHg | 79±10 | 78±11 † |
| Total cholesterol, mmol/L | 5.17±0.85 | 4.99±0.85 † |
| Triglycerides, mmol/L | 1.25 (0.86, 1.75) | 1.17 (0.82, 1.63) † |
| HDL-C, mmol/L | 1.50±0.41 | 1.53±0.39 |
| LDL-C, mmol/L | 3.03±0.78 | 2.87±0.72 † |
| Creatinine, µmol/L | 68.07±17.68 | 71.60±30.94 † |
| Fasting blood glucose, mmol/L | 5.83±1.11 | 5.83±1.11 |
| Hemoglobin A1c, % | 5.9±0.6 | 5.9±0.7 |
| hsCRP, ng/mL | 479 (264, 836) | 511 (270, 1040) |
| Medical history, n (%) |  |  |
| Hypertension | 356 (100) |  |
| Dyslipidemia | 239 (67.1) | 246 (69.1) |
| Diabetes mellitus | 68 (19.3) | 70 (25.3) |
| Current Smoking, n (%) | 38 (10.8) | 34 (9.6) |
| Medication, n (%) |  |  |
| Antihypertensive drugs | 350 (98.3) | 341 (95.8) |
| Calcium channel blockers | 236 (66.3) | 242 (68.0) |
| ARBs/ACEIs | 238 (66.9) | 222 (62.4) |
| Aldosterone antagonists | 9 (2.5) | 11 (3.1) |
| Lipid lowering drugs | 126 (35.4) | 153 (43.0) |
| Anti-diabetic drugs | 32 (9.0) | 41 (11.5) |
| baPWV, cm/seconds | 1607 (1441, 1791) | 1606 (1444, 1823) |
| Flow-mediated vasodilation, % | 4.6 (2.9, 6.4) | 4.5 (2.8, 6.9) |

†P<0.05 vs Baseline

HDL-C indicates high-density lipoprotein cholesterol; LDL-C, low-density lipoprotein cholesterol; ARB, angiotensin receptor blocker; ACE, angiotensin converting enzyme inhibitor; baPWV; brachial ankle pulse wave velocity; hsCRP, C-reactive protein.

**Supplementary Table 4.** Clinical Characteristics at Baseline and after 3-year Follow-up in Subjects with Hypertension who Had Exercise Habit at Baseline

|  | Stop exercise group | | Contentious regular exercise group | |
| --- | --- | --- | --- | --- |
| Variables | Baseline  (n=102) | 3 years  (n=102) | Baseline  (n=254) | 3 years  (n=254) |
| Age, yr | 64±9 |  | 64±8 |  |
| Men, n (%) | 57 (55.9) |  | 142 (55.9) |  |
| Body mass index, kg/m^2^ | 24.9±3.4 | 24.7±3.2 | 24.2±3.1 | 24.1±3.3 |
| Heart rate, bpm | 71±12 | 68±12† | 71±11 | 69±13 † |
| Systolic blood pressure, mmHg | 136±15 | 133±15 † | 134±15 | 135±15 |
| Diastolic blood pressure, mmHg | 79±10 | 77±11 † | 80±11 | 78±11† |
| Total cholesterol, mmol/L | 5.17±0.80 | 4.89±0.72 † | 5.17±0.85 | 5.04±0.88 † |
| Triglycerides, mmol/L | 1.30 (0.91, 1.76) | 1.15 (0.82, 1.50) † | 1.23 (0.85, 1.73) | 1.19 (0.82, 1.64) † |
| HDL-C, mmol/L | 1.50±0.41 | 1.50±0.39 | 1.53±0.41 | 1.53±0.41 |
| LDL-C, mmol/L | 3.03±0.83 | 2.82±0.67 † | 3.03±0.78 | 3.00±0.75 † |
| Creatinine, µmol/L | 68.07±17.68 | 74.26±46.85 | 68.07±17.68 | 70.72±20.33 † |
| Fasting blood glucose, mmol/L | 5.94±1.22 | 6.00±1.44 | 5.77±1.05 | 5.72±0.94 * |
| Hemoglobin A1c, % | 5.9±0.7 | 6.0±0.8† | 5.9±0.6 | 5.9±0.6 |
| hsCRP, ng/mL | 404 (197, 791) | 470 (235, 1045) | 489 (282, 908) | 525 (284, 1040) |
| Medical history, n (%) |  |  |  |  |
| Hypertension | 102 (100) |  | 254 (100) |  |
| Dyslipidemia | 70 (68.6) | 66 (64.7) | 169 (66.5) | 180 (70.9) |
| Diabetes mellitus | 23 (22.6) | 22 (21.5) | 45 (17.9) | 48 (18.9) |
| Current Smoking, n (%) | 15 (14.9) | 13 (12.8) | 23 (9.2) | 21 (8.3) |
| Medication, n (%) |  |  |  |  |
| Antihypertensive drugs | 102 (100) | 96 (94.1) | 248 (97.6) | 245 (96.5) |
| Calcium channel blockers | 72 (70.6) | 67 (65.7) | 164 (64.6) | 175 (68.9) |
| ARBs/ACEIs | 62 (60.8) | 62 (60.8) | 176 (69.3) | 160 (63.0) |
| Aldosterone antagonists | 5 (4.9) | 5 (4.9) | 4 (1.6) | 6 (2.4) |
| Lipid lowering drugs | 42 (41.2) | 52 (51.0) | 84 (33.1) | 101 (39.8) |
| Anti-diabetic drugs | 12 (11.8) | 17 (16.7) | 20 (7.9) | 24 (9.5) |
| baPWV, cm/seconds | 1652 (1311, 1816) | 1667 (1438, 1864) | 1567 (1440, 1780) | 1590 (1444, 1796) |

*P<0.05 vs Control group, †P<0.05 vs Baseline

HDL-C indicates high-density lipoprotein cholesterol; LDL-C, low-density lipoprotein cholesterol; ARB, angiotensin receptor blocker; ACE, angiotensin converting enzyme inhibitor; baPWV; baPWV, brachial ankle pulse wave velocity; hsCRP, high sensitivity C-reactive protein.

**Supplementary Table** **5.** Changes in Clinical Characteristics of Patients in Two Groups of Exercise Habit

| Variables | Total | Stop exercise | Regular exercise | P value |
| --- | --- | --- | --- | --- |
| Changes in body mass index, kg/m^2^ | -0.2±1.6 | -0.2±1.9 | -0.1±1.4 | 0.635 |
| Changes in heart rate, bpm | -2.0±11.7 | -2.5±11.7 | -1.8±11.7 | 0.591 |
| Changes in systolic blood pressure, mmHg | -0.1±15.7 | -2.0±15.7 | 0.7±15.6 | 0.138 |
| Changes in diastolic blood pressure, mmHg | -1.4±10.3 | -2.0±10.0 | -1.2±10.5 | 0.521 |
| Changes in total cholesterol, mmol/L | -0.18±0.75 | -0.26±0.82 | -0.14±0.71 | 0.185 |
| Changes in triglycerides, mmol/L | -0.06 (-0.34, 0.19) | -0.15 (-0.39, 0.14) | -0.03 (-0.32, 0.24) | 0.107 |
| Changes in HDL-C, mmol/L | 0.01±0.21 | 0.02±0.23 | 0.01±0.20 | 0.592 |
| Changes in LDL-C, mmol/L | -0.14±0.70 | -0.22±0.81 | -0.11±0.65 | 0.203 |
| Changes in creatinine, µmol/L | 4.42±42.43 | 6.19±38.90 | 2.65±8.84 | 0.184 |
| Changes in fasting blood glucose, mmol/L | -0.01±0.93 | 0.09±0.98 | -0.06±0.91 | 0.199 |
| Changes in hemoglobin A1c, % | 0.05±0.5 | 0.15±0.6 | 0.01±0.4 | 0.047 |
| Changes in hsCRP, ng/mL | 39 (-168, 353) | 39 (-108, 328) | 41 (-182, 362) | 0.571 |
| Changes in baPWV, cm/seconds | 24.5 (-125, 144) | 1.8 (-123, 148) | 29.5 (-128, 144) | 0.544 |

HDL-C indicates high-density lipoprotein cholesterol; LDL-C, low-density lipoprotein cholesterol; hsCRP, high sensitivity C-reactive protein; baPWV, brachial ankle pulse wave velocity.

**Supplementary Figure**

**Supplementary Figure 1.**

**
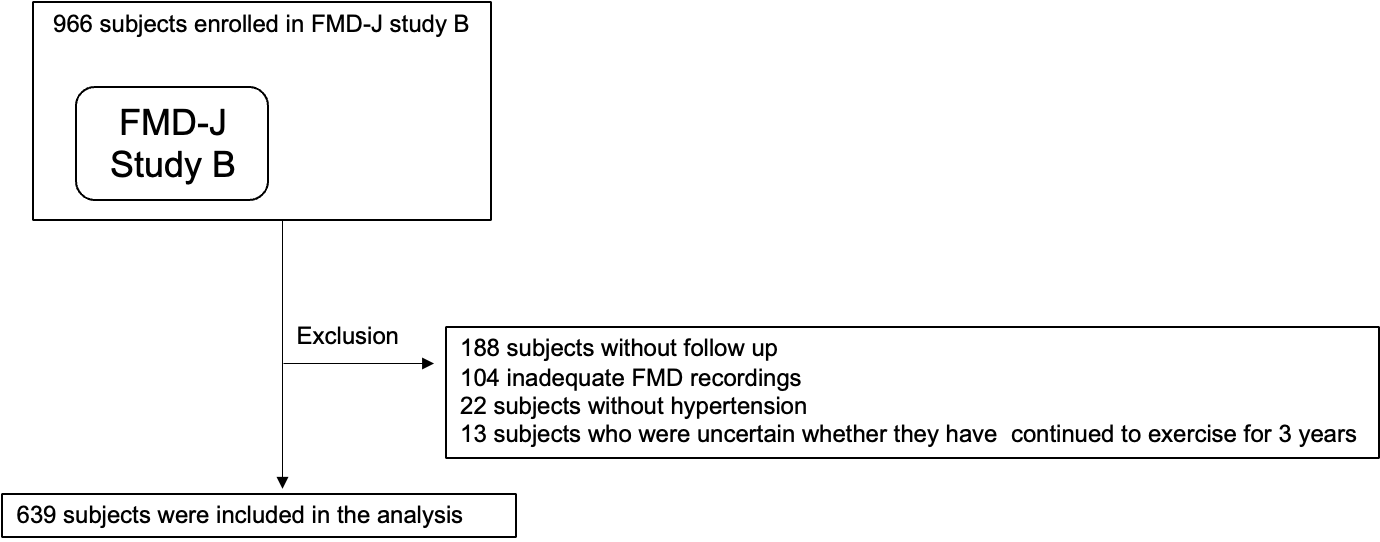
**

**Supplementary Figure 1.** Flow chart of the study design.

FMD-J indicates flow-mediated dilation Japan.
